# Comparison of endoscopic based diagnosis with *Helicobacter* urease test for *Helicobacter pylori* infection

**DOI:** 10.1186/s13104-016-2237-6

**Published:** 2016-08-30

**Authors:** N. A. Adu-Aryee, L. Aabakken, F. Dedey, J. Nsaful, W. Kudzi

**Affiliations:** 1 Department of Surgery, School of Medicine and Dentistry, College of Health Sciences, P.O. Box GP 4236, Accra, Ghana; 2 Division of Gastroenterology, University of Oslo, Oslo, Norway; 3 Centre for Tropical Clinical Pharmacology and Therapeutics, School of Medicine and Dentistry, College of Health Sciences, P.O. Box GP 4236, Accra, Ghana

**Keywords:** *Helicobacter pylori*, *Helicobacter* urease test, Endoscopic diagnosis

## Abstract

**Background:**

*Helicobacter pylori* is an important risk factor for gastritis, peptic ulcers and gastric cancer. The prevalence in developed countries is lower than 40 % but higher than 80 % in some developing countries. It is 75 % in Ghana. The *Helicobacter* urease test (HUT) is performed at endoscopy and gives an accurate diagnosis. The HUT is not routinely done at our facility and presumption of *H. pylori* is made based on endoscopic findings and *H. pylori* eradication prescribed, as the incidence in the general population is presumed high. Is this endoscopic diagnosis sufficient for diagnosing and treating *H. pylori*? We aimed to assess the feasibility of an endoscopic based *H. pylori* diagnosis and its accuracy using a HUT as the gold standard in consecutive patients.

**Methods:**

Seventy-six consecutive adult patients with dyspepsia were assessed by upper gastrointestinal endoscopy. A clinical diagnosis of *H. pylori* or not was made. Biopsy samples were collected for HUT. *H. pylori* was diagnosed if HUT was positive. The results were then compared.

**Results:**

Median age of patients was 45.0 years. *H. pylori* prevalence detected by HUT was 51.3 % (95 % CI 40.0–63.0). Sensitivity of endoscopic diagnosis of *H. pylori* was 71.8 % (95 % CI 55.1–85.0) and specificity was 37.8 % (95 % CI 22.5–55.2). There was no association between clinical findings (73.7 %) and HUT (26.3 %) (OR = 0.80; [95 % CI 0.24–2.64], p = 0.682). There was also no association between endoscopic diagnosis (71.8 %) and HUT (28.2 %), (OR = 1.55; 95 % CI 0.59–4.06, p = 0.373).

**Conclusion:**

*Helicobacter pylori* infection was not as high as that published in earlier reports. The endoscopic diagnosis alone is not sufficient to make a diagnosis of *H. pylori*.

## Background

*Helicobacter pylori* (*H. pylori*) infection is the most common cause of bacterial infection worldwide [1, 2] infecting more than 50 % of the world’s population predominantly in the developing countries [3]. *H. pylori* is an important risk factor for gastritis, peptic ulcers, and gastric cancer likely due to the extensive inflammation in the stomach [3, 4]. *H. pylori* has been associated with the development of two forms of cancer and has led to the World Health Organization (WHO) classifying it as the only bacterial Class I carcinogen. Unless treated, colonization usually persists for life, indicating that *H. pylori* is well adapted to the gastric environment.

Prevalence of *H. pylori* infection varies among countries and within different racial groups within the same country. The highest rates of infection are generally associated with low socio‐economic status, crowding, poor sanitation and unclean water supplies [5].

In the developed countries the prevalence of *H. pylori* is lower than 40 % but in some developing countries more than 50 % of children are infected by the age of 10 years with prevalence of infection rising to more than 80 % in young adults [6]. In Ivory Coast, 55 % of children aged <10 years have been reported to be infected, while in northern Nigeria and Gambia, 50 % of children under 5 years are infected [7]. A recent study in Soweto found 46 % of children at 1 year and 100 % of children at 12 years to be infected with *H. pylori* [7]. The prevalence of *H. pylori* in dyspeptic patients has been reported to be 75 % in Ghana [3, 4], 91.3 % in Ivory Coast [8] and 72–91 % in Nigeria [9, 10].

The diagnosis of *H. pylori* infection relies on the various testing methods which may be invasive or non-invasive. There are invasive techniques such as histological examination, bacterial cultures, rapid urease test, use of deoxyribonucleic acid probes, and polymerase chain reaction (PCR) analysis all of which require an endoscopy and a biopsy. Non-invasive techniques such as urea breath tests, immunoglobulin G and M serology, Stool antigen test, gastric juice PCR, and urinary excretion of N15 ammonia do not require endoscopy. These various methods have been found to be sensitive and specific [1] but require skilled persons to perform them correctly. *H. pylori* urease test is fast, simple and does not need highly experienced laboratory staff to accurately identify *H. pylori* infection in the endoscopy unit within few hours of the procedure [11].

Epigastric pain, dyspepsia, haematemesis and melaena have been the commonest reasons for endoscopy. Chronic duodenal ulcer, acute gastritis, duodenitis and oesophagitis were the commonest diagnoses in Korle Bu Teaching Hospital from January 1995 to December 2002 [3]. *H. pylori* plays an important role in the aetiopathogenesis of peptic ulcer disease among Ghanaians [3, 4]. Previous studies in Korle Bu Teaching Hospital using urease test confirmed positive results in 75 % of all biopsy specimen [3]. Early diagnosis and treatment can lead to the eradication of *H. pylori* which will reduce *H. pylori* related peptic ulcer disease. *H. pylori* testing schemes in Accra are diverse and non-systematic. Upper gastrointestinal (GI) endoscopy is an established method of investigation in Korle Bu Teaching Hospital for *H. pylori* diagnosis using antral biopsies for histology. However, it is not routinely performed. The current practice in Korle Bu Teaching Hospital involves making a diagnosis based on endoscopic findings. Occasionally biopsies may be taken for histologic conformation of *H. pylori* infection.

This study determined the current incidence of *H. pylori* infection in Korle Bu Teaching Hospital and assessed the feasibility of an endoscopic based *H. pylori* diagnosis and its accuracy using a *Helicobacter* urease test (HUT) as a gold standard in a cross-sectional study of patients.

## Methods

The study was conducted at the Endoscopy Unit of Korle Bu Teaching Hospital. It is a referral hospital with over 1800 beds for in-patients and has several specialist clinics, wards, pharmacies and reference laboratories. The study protocol was approved by Ethical and Protocol Review Committee of the School of Medicine and Dentistry, University of Ghana and all participating patients or their relatives provided written informed consent.

Seventy-six patients between 16 and 81 years referred for upper gastrointestinal endoscopy with dyspeptic symptoms were recruited for this study. Baseline bio-data were obtained from all patients and diagnosis of gastric disorders were made based on the presence of atrophic mucosa, oedematous mucosa, red spots or streaks and erosions suggesting inflammation and active or healing ulcers. Endoscopic diagnosis was made at the discretion of the endoscopist. Gastric antral mucosal biopsies of adequate size were then taken for *H. pylori* urease testing (HelicotecUT^®^ Plus, Emergo Europe). In regions of atrophic gastritis, the biopsies were taken more proximally.

### *Helicobacter* urease test (HelicotecUT^®^ Plus)

Biopsy samples, approximately 2–3 mm each were taken from the antralgastric mucosa and placed on the yellow colored well containing urea and a pH indicator. The production of the urease enzyme by *H. pylori* results in the decomposition of urea into bicarbonate and ammonia which causes the pH to rise and the colour of the dot to change from yellow to red or pink. Positive results were read within 5 to 30 min. Samples that were weakly positive took up to 1 h to develop and no colour change at 1 h was regarded negative.

### Statistical analysis

All data were entered into Statistical Package for Social Science (ver.17.0; SPSS, Chicago, IL) and imported into Stata™ version 10 (StataCorp, College Station, Texas, United States) for statistical analyses. Data were summarized as frequencies and proportions.

## Results

A total of 76 patients were recruited for the study of which 43 (56.6 %) were females and 33 (43.4 %) were males. The median age of the study participants was 45.0 years [IQR 30.5–63.5]. A vast majority of the study subjects (88.2 %, n = 67) were from urban areas and 9 (11.8 %) were from rural areas. Severity of symptoms was assessed to be mild in 9 (12.0 %) and moderate to severe in 66 (88 %) of the patients. Endoscopic diagnosis was positive for *H. pylori* in 51 patients (67.1 %). However, the overall prevalence of *H. pylori* using the HUT among the recruited patients was 51.3 % [95 % CI 40.0–63.0].

Table 1 shows the distribution of the various symptoms reported by the patients. A total of 39 patients recruited in the study were positive for *H. pylori* urease test. The most reported symptom by patients was epigastric pain (67.1 %, n = 51). Among these patients reporting with epigastric pain, 74.4 % (n = 29) tested positive for *H. pylori* urease test. For patients reporting with dyspepsia 23.1 % (n = 9) were positive for HUT (Table 1).

**Table 1:** Frequency of symptoms of patients attending endoscopy clinic at the Korle Bu Teaching Hospital in Accra, Ghana

Symptom	*H. pylori* urease test results		Endoscopic diagnosis results	
	Positive N = 39 n, %^a^	Negative N = 37 n, %^a^	Positive N = 51 n, %^a^	Negative N = 25 n, %^a^
Epigastric pain	29 (74.4)	22 (59.5)	35 (68.6)	16 (64.0)
Nausea	3 (7.7)	0 (0)	2 (3.9)	1 (4.0)
Vomiting	1 (2.6)	0 (0)	1 (2.0)	0 (0)
Dyspepsia	9 (23.1)	5 (13.5)	7 (13.7)	7 (28.0)
Chest pain	7 (17.9)	6 (16.2)	9 (17.6)	4 (16.0)
Hematemesis	4 (10.3)	8 (21.6)	8 (15.7)	4 (16.0)
Malena	3 (7.7)	9 (24.3)	8 (15.7)	4 (16.0)
Regurgitation	1 (2.6)	4 (10.8)	3 (5.9)	2 (8.0)

^a^  %s may not add up to 100 as one patient be diagnosed with more than one endoscopic finding

Table 2 shows the various endoscopy findings. The most frequent endoscopy finding was gastritis (59.2 %, n = 45). Seventeen patients (22.4 %) had normal endoscopy results. A total of 26 (66.7 %) of the patients who tested positive to HUT were diagnosed with gastritis whilst 8 (20.5 %) were diagnosed with gastric ulcer (Table 2).

**Table 2:** Frequency of endoscopy findings of patients attending endoscopy clinic at the Korle Bu Teaching Hospital in Accra, Ghana

Endoscopic finding	*H. pylori* urease test results		Endoscopic diagnosis results	
	Positive N = 39 n, %^a^	Negative N = 37 n, %^a^	Positive N = 51 n, %^a^	Negative N = 25 n, %^a^
Gastritis	26 (66.7)	19 (51.4)	40 (78.4)	5 (20.0)
Gastric ulcer	2 (5.1)	4 (10.8)	6 (11.7)	0 (0)
Duodenal ulcer	6 (15.4)	4 (10.8)	10 (19.6)	0 (0)
Gastric polyp	0 (0)	1 (2.7)	0 (0)	1 (4.0)
Normal	6 (15.4)	11 (29.7)	2 (3.9)	15 (60.0)
Candidiasis	0 (0)	1 (2.7)	1 (2.0)	0 (0)
Reflux esophagitis	1 (2.6)	0 (0)	1 (2.0)	0 (0)
Oesophageal stricture	0 (0)	1 (2.7)	1 (2.0)	0 (0)

^a^  %s may not add up to 100 as one patient be diagnosed with more than one endoscopic finding

Table 3 shows the association between the various exposure characteristics and HUT. In the univariate analysis, age grouping, dwelling status, endoscopy findings and endoscopy *H. pylori* diagnosis were not associated with HUT (p > 0.05). In the multivariate analysis, gender and symptom severity were associated with *H. pylori* urease test results. The odds of a female showing positive test results for HUT was 2.73 times compared with males (OR 2.73 [95 % CI 1.02–7.33], p = 0.0460) although this association is marginal (Table 3). Symptom severity was associated with HUT results such that the odds of a patient reporting with moderate to severe symptoms having a positive HUT test results was 12.06 times (OR 12.06 [95 % CI 1.38–105.59], p = 0.024) compared with patients who reported with mild symptoms (Table 3).

**Table 3:** Factors associated with positive *H. pylori* urease test

Characteristic	*H. pylori* urease test		Crude OR [95 % CI]	p value	Adjusted OR [95 % CI]	p value
	Positive n, %	Negative n, %				
Gender						
Female	26 (66.7)	17 (46.0)	2.35 [0.93–5.95]	0.069	2.73 [1.02–7.33]	0.046
Male	13 (33.3)	20 (54.1)	Ref		Ref	
Age group						
<20	1 (2.6)	1 (2.7)	1.33 [0.07–24.32]	0.846	–	–
20–29	9 (23.1)	8 (21.6)	1.50 [0.41–5.43]	0.537	–	–
30–39	9 (23.1)	5 (13.5)	2.40 [0.60–9.67]	0.218	–	–
40–49	4 (10.3)	7 (18.9)	0.76 [0.17–3.42]	0.723	–	–
50–59	7 (18.0)	4 (10.8)	2.33 [0.52–10.48]	0.269	–	–
≥60	9 (23.1)	12 (32.4)	Ref			
Dwelling						
Rural	4 (10.3)	5 (13.9)	0.71 [0.13–3.63]	0.629	–	–
Urban	35 (89.7)	31 (86.1)	Ref			
Symptom severity						
Moderate/severe	38 (96.4)	28 (77.8)	10.86 [1.29–94.05]	0.009	12.06 [1.38–105.59]	0.024
Mild	1 (2.6)	8 (22.2)	Ref		Ref	
Endoscopy finding						
Abnormal	28 (73.7)	28 (77.8)	0.80 [0.24–2.64]	0.682	–	–
Normal	10 (26.3)	8 (22.2)	Ref			
Endoscopic diagnosis						
Positive	28 (71.8)	23 (62.2)	1.55 [0.59–4.06]	0.373	–	–
Negative	11 (28.2)	14 (37.8)	Ref			

Table 4 shows the various medications that the patients were taking and how it related to the *H. pylori* status as detected by HUT and by endoscopic diagnosis. 18 % of patients who admitted to taking PPI’s were HUT positive while 51 % were negative.

**Table 4:** Comparison of medications with *H. pylori* urease test and with the endoscopic diagnosis

Medication	HUT results		Endoscopic findings	
	Positive (%)	Negative (%)	Positive (%)	Negative (%)
PPI				
Yes	7 (18.0)	19 (51.4)	14 (27.5)	12 (48.0)
No	32 (82.0)	18 (48.6)	37 (72.5)	13 (52.0)
Antacid				
Yes	9 (23.1)	8 (21.6)	14 (27.5)	3 (12.0)
No	30 (76.9)	29 (78.4)	37 (72.5)	22 (88.0)
H_2_ blocker				
Yes	1 (2.6)	0 (0)	1 (2.0)	0 (0)
No	38 (97.4)	37 (100)	50 (98.0)	25 (100)
NSAID				
Yes	13 (33.3)	8 (21.6)	17 (33.3)	4 (16.0)
No	26 (66.7)	29 (78.4)	34 (66.7)	21 (84.0)

Diagnostic yield of endoscopic diagnosis is 55.3 %. Using endoscopic diagnosis as the index test and HUT as the reference standard, the sensitivity and specificity of the index test were 71.8 % [95 % CI 55.1–85.0] and 37.8 % [95 % CI 22.5–55.2] respectively. Further analysis indicated that the area under the receiver operated characteristics curve was 0.55 [95 % CI 0.44–0.65] indicating that the endoscopic diagnosis test do not predict *H. pylori* status (as determined by HUT) better than chance. After excluding those on PPI, diagnostic yield was 58 %, sensitivity of 75.03 [95 % CI 56.6–88.5] and specificity of 38.8 % [95 % CI 13.9–68.4]. The area under the receiver operated characteristics curve was 0.51 [95 % CI 0.37–0.66] implying that in patients who have not been exposed to PPI endoscopic diagnosis test do not predict *H. pylori* status.

## Discussion

The most common symptoms leading to referral for upper GI endoscopy were epigastric pain, dyspepsia, upper gastrointestinal bleeding (haematemesis and melaena) and chest pain respectively which is the same as that reported in earlier publications [3]. The current study found the incidence of *H pylori* infection in Korle Bu Teaching Hospital to be 51.3 % based on the HUT. The prevalence is much lower than that quoted in other studies from West Africa [3, 4, 9] which have shown a high prevalence of *H. pylori*. Previous studies in Korle Bu Teaching Hospital reported an incidence of 75 % [3, 4]. However, a recent publication also reports an *H. pylori* prevalence of 69.7 % (1999) and 45.2 % (2012) in Accra [12]. This suggests a change in *H. pylori* prevalence with time.

Gastritis (59.2 %) was the most common identifiable lesion at endoscopy in this study. One study done in Tanzania with *H. pylori* prevalence of 65 % also reported gastritis as the most common finding and gastritis and duodenal ulcer were statistically associated with *H. pylori*. 72 % of gastritis and 89.5 % of duodenal ulcers were *H. pylori* positive [13]. Based on previous prevalence studies, it has been established that the finding of a non-bleeding duodenal ulcer has a positive predictive value of over 90 % for *H. pylori* diagnosis, making confirmatory tests not mandatory. However, the positive predictive values for gastric ulcers, bleeding duodenal ulcers and perforated duodenal ulcers are lower and confirmatory tests would be necessary [14].

Nearly 58 % of gastritis and 60 % of duodenal ulcers tested positive for *H. pylori,* while 33 % of gastric ulcers were also positive. Interestingly our study did not find any positive association between duodenal ulcers, gastric ulcers or gastritis and a positive HUT. This may be explained by the fact that, among the 10 patients with duodenal ulcer, 3 of the 4 patients who were HUT negative admitted to using PPI’s. While of the 6 patients diagnosed with gastric ulcer, 3 of the 4 who had negative HUT admitted to using PPI’s. Cohen et al. [14] have reported that the use of PPI’s may lead to unreliable *H pylori* diagnosis.

Moderate to severe symptoms were associated with HUT results such that the odds of a patient reporting with moderate to severe symptoms of having a positive HUT test result was 12.06 times (OR 12.06 [95 % CI 1.38–105.59], p = 0.024) compared with patients who reported with mild symptoms. This means endoscopy with *H. pylori* diagnosis and treatment is mandatory in these patients whereas those with mild symptoms may be managed without *H. pylori* testing.

The gold standard recommended by the World Gastroenterology Organisation Global Guidelines, 2010 is endoscopy and rapid urease test which may not be readily available or cost effective in developing countries. They recommend then that in resource limited settings with a high prevalence, the decision to treat may be based on the assumption that *H. pylori* is present. This has been the practice over the years, however, recent development has seen the introduction of several endoscopy set ups in both public and private facilities. This has brought with it increased opportunities for the accurate diagnosis and treatment of *H. pylori*. Accurate diagnosis and treatment is necessary if the prevalence is not as high as previously reported. The empirical treatment with antibiotics based on endoscopic diagnosis and assumption of high prevalence should be discouraged. Efforts should be made to make an accurate diagnosis of *H. pylori* before treatment. Where the HUT is not available other tests should be done such as histological diagnosis or stool for *H. pylori* antigen. The lower prevalence we report may be due to improving standards of living and sanitation in Ghana as compared to previous studies published several years ago also using HUT [3, 4]. In addition the vast majority of our study subjects were from urban areas where the standard of living is higher. However, the frequent use of antibiotics may also contribute to the relatively low prevalence being reported recently [12]. Also the recent use of PPI’s may contribute to the low prevalence. Of all the patients who admitted to taking PPI’s only 18 % had positive HUT as compared to 51 % who tested negative. While for those who denied PPI use, 82 % had positive HUT and 49 % were negative. This supports the fact that recent use of PPI’s may result in falsely negative HUT results [14].

With regards to the endoscopic diagnosis this study reports a diagnostic yield of 55.3 % with a sensitivity and specificity of 71.8 % [95 % CI 55.1–85.0] and 37.8 % [95 % CI 22.5–55.2] respectively, indicating that the endoscopic diagnosis does not predict *H. pylori* status (as determined by HUT) better than chance. After excluding those on PPI, analysis resulted in a diagnostic yield of 58 %, sensitivity of 75.03 [95 % CI 56.6–88.5] and specificity of 38.8 % [95 % CI 13.9–68.4]. The value of endoscopic diagnosis of *H. pylori* is unclear. The value of the HUT in the patients who have recently been on PPIs and antibiotics maybe limited. In this case treatment based on the endoscopic diagnosis should be considered as recommended by WHO. On the other hand in treatment naive patients, the HUT maybe more reliable and treatment can be prescribed when indicated.

It is known that certain endoscopic mucosal features indicate *H. pylori* infection such as atrophic changes, rugal hyperplasia, oedema, spotty erythema, linear erythema and haemorrhage, amongst others. However, recognition and diagnosis depends on the experience of the endoscopist. Several studies including that by Khaloo et al. (41.8 %) [15] and Redeen et al. (43–53 %) [16] have reported a low diagnostic yield. Khazuhiro et al. however, published a relatively higher diagnostic yield in the *H. pylori*-uninfected (88.9 %) but lower in *H. pylori*-infected (62.1 %) and in the *H. pylori*-eradicated (55.8 %) patients [17]. This current study, like others, does not support endoscopic diagnosis of *H. pylori* as a standard of care.

Magnifying endoscopy affords the opportunity to make an accurate endoscopic diagnosis of *H. pylori* based on the surface structure of the gastric mucosa and has sensitivity and specificity of 100 and 92.7 % respectively for *H. pylori* infected mucosa [18]. This facility is not available at many endoscopy centers worldwide and not in Ghana and other developing countries. It may now be cost effective to make an accurate diagnosis before treatment so as to cut down cost on antibiotics, its side effects and reduce antibiotic resistance.

The obvious advantage of the rapid urease test in our environment is that it is inexpensive (less than $10/test), does not require any technical expertise and gives rapid results within an hour. It is also reliable with the sensitivity of *Campylobacter*-like organism (CLO) test 75–98 % and specificity 95–100 % [14]. This will improve the accurate diagnosis especially where endoscopic expertise is not certain.

With histologic diagnosis the disadvantages are sampling errors, observer variations, high cost and long duration of processing. There is also an additional delay in our facility as the patient is responsible for getting the specimen to the pathologist and getting the report back to the physician.

### Limitation

One of the limitations of our study is taking antral biopsy samples. We also did not have the sufficient resources to recruit more patients, which may have improved the statistical power of our studies. This study was also undertaken in a single medical center, Korle Bu Teaching Hospital, which may not be a general representation of Ghana although the patients came from variety of ethnic groups and socioeconomic backgrounds.

## Conclusion

The incidence of *H. pylori* infection in Ghana may be lower than previously reported due to changing socioeconomic factors, increased PPI and antibiotic use. To determine the true incidence of *H. pylori* infection in Ghana a larger study cutting across all the socio-economic classes needs to be done on a treatment naive population. The endoscopic diagnosis alone of *H. pylori* infection is not sufficient as it has a low sensitivity and specificity and depends on the experience of the endoscopist. The rapid HUT is simple and inexpensive and should be introduced routinely at all endoscopy centers in Ghana and other developing countries. Endoscopists at these centers should be educated on its interpretation based on patient’s drug history.

## Abbreviations

*H. pylori*: *Helicobacter pylori*
HUT: *Helicobacter* urease test
CI: confidence interval
CLO: *Campylobacter*-like organism
OR: odd ratio
DNA: deoxyribonucleic acid
PCR: polymerase chain reaction
UBT: urea breath tests
KBTH: Korle Bu Teaching Hospital
